# Lesbian Community and Activism in Britain 1940s–1970s: An Interview with Cynthia Reid

**DOI:** 10.1080/00918369.2021.1996098

**Published:** 2021-11-01

**Authors:** Katherine A. Hubbard

**Affiliations:** Department of Sociology, University of Surrey, Guildford, UK

**Keywords:** British lesbian history, activism, microhistory, Minorities Research Group/Kenric, queer history, oral history

## Abstract

In this article, I provide a micro(oral)history of Cynthia Reid, one of only five women who founded the Minorities Research group—the first known lesbian organization in Britain, in 1963. Such activism paved the way for further lesbian liberatory action and the group did a great deal to combat the isolation experienced by many queer women across the country. They provided social opportunities as well as advice, and made more public calls for greater social acceptance. The group has been central to the interests of 20th-century queer historians, especially as the Minorities Research Group also produced the first lesbian magazine in Britain *Arena Three*. As a microhistory Cynthia’s story informs many threads within queer history, including conceptualizations of masculinities, community, and change; while also challenging dominant notions that families and medical professionals were consistently unsupportive of queer people in the 1940s–1970s. In doing so, this article amplifies Cynthia’s story in a way that means not only does it contribute nuance as a micro(oral)history to the broader field of queer scholarship but it also acts as a resource to stimulate further research.

## Introduction

This article presents excerpts and contextualizing analysis of an interview with Cynthia Margaret Bernice Reid. Cynthia was one of the founding members of the first official (known) lesbian organization in Britain—the Minorities Research Group in 1963. Following this she participated in the formation of their splinter group Kenric, which is still active today and is now the longest ever running group of its kind in Britain. Cynthia’s story provides a microhistory from which to consider queer history broadly, and lesbian liberation specifically. Such a history gives particular insight into lesbian lives and activism between 1940 and 1970s. Threads which run throughout the proceeding oral history illuminate prominent aspects of women’s queer history including conceptualizations of masculinities, community, and change (eg, see Boyd, 2003; Gardiner, 2003; Jennings, 2007a, 2007b; Traies, 2018). This article aims to demonstrate the value in individual histories for wider historical inquiry as well as provide a primary source for those interested in queer history and scholarship.

In providing an oral history in this article, it is possible to see threads and themes from broader queer history in the example of Cynthia Reid’s life. It also reveals the retrospective experiences of pioneering lesbian activism in the 1960s. Like a lot of history, individual’s stories are often what are most powerful, and so here I present Cynthia’s mostly in her own words, albeit with biographical and contextualizing information.^1^ An oral history, unlike other forms of interview, has particular worth in creating histories with individuals who experienced significant events without imposing as much expectation and interpretation. Oral histories have been said to be “the first kind of history” (Thompson, 2000) but largely fell out of academic favor until the 1970s when they saw a resurgence as a key method of social history. Rather than the conventional elitist historical story-telling, oral history instead promotes the stories of those “from below.” That is the otherwise forgotten and untold story-tellers, whose voices often get ignored, analyzed, or at the very least, interpreted/translated.

This approach has emphasized revealing “hidden histories” and valuing memory. For this reason, oral histories have been favored by feminist historians and those concerned with the histories of lesbian, gay, bisexual, trans and queer (LGBTQ) individuals (see Tooth Murphy, Wall, Vickers, & Severs, 2020).^2^ Feminist approaches and oral histories have a supportive relationship on the basis on their similar epistemological standpoints (Gluck & Patai, 1991). Marginalized voices have long been silenced in cultural histories, including LGBT histories (see Namaste, 2000) and so by naming and empowering those who share their stories it is possible to amplify their voices and remove invisibility. Open interview styles and approaches allows for more marginalized narratives to be able to be told, and this appears to be particularly prominent when thinking about lesbian history/herstory. A key example is *Boots of Leather, Slippers of Gold: The History of a Lesbian Community* by *Boots of Leather, Slippers of Gold: The History of a Lesbian Community* Lapovsky Kennedy and Davis (2014; see also Davis & Lapovsky Kennedy, 1986), and the oral histories around the establishment and continuation of the Lesbian Herstory Archives (see Nestle, 1993; Smith-Cruz et al., 2016). Like Nestle’s (1993) oral history interview with Mabel Hampton, this article reflects on the narrative that Cynthia has decided to remember, and is able to tell, but unlike most of the aforementioned examples, contributes to a British history (following in the footsteps of Summerskill, 2013; Traies, 2018). This micro(oral)history not only contributes the unique oral history of Cynthia Reid, who is thought to be only surviving founding member of the Minorities Research Group, but also provides further resources to the growing range of literature that provides materials of oral history in such a way that allows others to utilize the same source data (eg, Lewis, 2016; Oram & Turnbull, 2001; and the book *Inventing ourselves: Lesbian life stories*).

In concentrating on an oral history of one individual, this article also presents a form of microhistory. That is, a history which focuses in on a smaller, more niche subject, though microhistory has somewhat eluded definition (Lepore, 2001). By drawing in scope, it is possible to include more nuance, detail and contextualization of important specific events, periods or people which otherwise tend to be obscured. It is said to be an historical approach that concentrates more on the “everyday” and, much like those approaches that considered gender to a greater extent, became increasingly popular from the 1970s onward (Lüdke, 1989; Morgan, 2006). Microhistory, like oral history, also tends to place the researcher in the scene as a participant to the narrative in some way (Lepore, 2001, see Creet, 2014). This is why, throughout the article, I deliberately position myself as part of the conversation and include my own interjections and questions. I also recognize the intersubjectivity of being an “insider” in this interview (Tooth Tooth Murphy, 2020b), and my approach is particularly informed by feminist epistemological approaches (eg, Haraway, 1991; Oakley, 1981).^3^ This article therefore combines oral history and microhistory to form a micro(oral)history, with the aim to demonstrate how a single narrative can be used to (re)consider and reflect upon a wider cultural context. I argue that the experiences and actions of Cynthia Reid, as told in her own words, provide a fruitful addition to understanding broader mid-century queer British lesbian history.

The article is separated into three main sections that are structured to foreground Cynthia’s words; following this I provide a contextualized elaboration, which relates her story to relevant literature. The first section contains Cynthia’s reflections on her early life and youth from the 1940s–1950s. That is, her descriptions of her more “masculine pursuits” and interests as well as her family’s expectations. It includes her time at Cambridge University, the exploration of her sexuality, her masculinity and conceptualizations of sex. The second section charts her life in the 1960s. This was central period as it was then she began in earnest to work against the isolation facing queer women in Britain at the time. This section includes her experiences with the Homosexual Law Reform Society, the Minorities Research Group and Kenric. The third section considers her life from the 1970s onward and it is in this section that I argue Cynthia can be considered an activist, though she does not identify herself in this way. The conclusion considers Cynthia’s legacy and thoughts on the contributions this article is able to make to the literature, and future research endeavors.

## 1940s–1950s: Coming out

Cynthia Reid was born in 1935 in Leeds, England, to a close-knit family consisting of her mother, father, and her maternal grandmother who lived with them in her early childhood. She comes from a white, English, middle-class background. She was four years old when the Second World War broke out at which point the family moved “lock, stock and barrel” to her paternal grandparent’s house on the outskirts of Leeds so they, in Cynthia’s words: “didn’t have to lose me and I didn’t have to be evacuated.”

“I was much protected from the War, I think, I mean I took my gas mask to school like everybody else and we played aeroplanes in the bits of walls that had been taken after the air raids from the war effort and that sort of thing. So the war affected me to some extent but we didn’t have much bombing in Leeds”

“Playing aeroplanes” soon became more serious as it developed into a lifelong passion. At the age of 18 Cynthia qualified as a pilot at Yeadon Flying Club^4^ whilst also studying at Leeds Girls High School. In fact, being a pilot is very central to Cynthia’s identity. When I asked Cynthia how she identifies, she replied:

“I suppose lesbian. That’s what I’ve always been. The fact that I haven’t had a partner for the past x years, god knows how long now, that doesn’t matter but that’s how I would identify.”

**Katherine**: That doesn’t matter, no. Excellent, and is there anything else that’s important to you …

“I suppose what’s the important things I identify myself as? As a pilot first and foremost, I’ve been interested in aviation since I was that high [indicates low height] … I was, one day, going to have an aeroplane: G-CMBR. My initials CMBR … one day I might have an aeroplane that had CMBR on it, *and it does*.” (my emphasis)

At 19 Cynthia fulfilled her father’s ambition and went to Cambridge University on a scholarship initially to study Mathematics, later Mechanical Sciences.

“Before I was even conceived I was destined you know. I had to get to Cambridge. I went to Leeds Girls High and there I stayed until I was 19 and then finally got scholarships offered at Oxford and Cambridge and chose Cambridge because that was the destiny … my parents never, never doubted that … Yes, it was a ridiculous thing, because at the time … women at Oxford and Cambridge didn’t get degrees. They were allowed to study but they were not allowed a degree, until 1946.^5^ So he was pushing me towardsa University that wasn’t even going to give me a degree when I got to the end. As it happened they changed the rules and by the time I got there they did give degrees. So I did get my degree. I went there on Mathematics because no one had heard of a woman going up on what they called Mechanical Sciences at Cambridge, which is called engineering everywhere else, but yes, so I switched courses half way through … I was the only woman. In a faculty of 2000 men in engineering. I was the only woman.”

**Katherine**: Was that very challenging?

“Not to me. No, I never really thought about it, to be honest. No no, never really thought about it. By then I had my pilots license so I’d mixed and mixed in garages and all my upbringing was really, I suppose oriented towards learning car mechanics from my father, astronomy from my father, my poor mother who was an artist and a good needle woman and things like that, just didn’t interest me. So I was fortunate in the way that my own leanings coincided with what my father planned really.”

At Cambridge Cynthia continued to fly and also began to explore her attraction toward other women eventually establishing a lesbian relationship with another student. She traces back her first thoughts about her own sexuality and gender to an early age.

“The attraction towards women didn’t start until I was about 12 when I fell in love with the maths teacher at school. But knowing that I was, I suppose you’d call it being a tom boy in those days, that started I should think, the earliest memory was about 5 years old. When I had some grey shorts and a grey blazer and the next-door neighbour said ‘Oh I thought you were a little boy’ and I thought ‘Yes, I’m a little boy’ [laughter] … So I never really had any concept of marriage and children really. No no, it never crossed my mind. Everything was revolved around maths and astronomy and changing car wheels and things like that … I don’t know if my mother had any input to that. She was, I suppose, not a very maternal woman, she was, she never pushed maternal things onto me. I got given a doll and a pram once but it was much more important that I was also given roller-skates and a football and a cricket bat and things like that. So I was much more interested in masculine pursuits I suppose, but not because they were masculine, but just because they were the things I found interesting.”

“In those days, all school children had crushes on school teachers and usually school teachers were the same sex so boys would have crushes on their masters and we girls would have crushes on mistresses.^6^ There was never anything that was thought odd, you had a passion, for a particular school teacher, or in some case on a senior prefect, you know. My mother grew up in age where they were called ‘special friendships’ and she had a special friendship with a Jewish woman from the age of 18 at work, you know. This woman helped her settle when she’s come back from American from the War … . And women had these special friendships and I suppose men did, you know best buddies. But nobody ever, it wasn’t a sexual thing and nobody thought anything about it. So no, it didn’t arise until I was in my 20s really and came out.”

At the time of her “coming out” there was a lack of publicly available language to describe lesbianism (Jennings, 2007b). Therefore, Cynthia didn’t have the language to enable her to label her attraction to women. This led to her feeling that the answer would be for her to change sex in order to continue the relationship with her girlfriend.

“When I did [come out] I thought I had to change sex. Because I’d never heard of homosexuality. I was … 21 years old by then in my second year at Cambridge and I developed this sexual relationship with another student. And I didn’t know what to do about it because I had never come across anything you know, sexual like that before. I’d had sexual relationships of a sort with boys, you know heavy petting that kind of thing but I was never emotionally involved with them. So, it didn’t really impinge upon me at all. And I was totally confused really. And I, I’d wrote to my parents and said, you know: ‘There’s this friend of mine and I think I shall have to change my sex so we can get married.’ And they contacted the local doctor, you know, they were quite supportive. They weren’t like ‘get out of my house’ sort-of-thing. They contacted the family doctor and he was out of his depth as well, he had no idea what to do. So he said he’d have to consult with some colleagues and eventually the answer came back ‘well you can’t change sex, there’s no such thing as changing sex, it’s probably a phase, she’ll grow out of it, don’t worry about it.’”^7^

“I think my parents contacted the college at Cambridge and I think it was accepted there that women did have relationships and I was sure it was accepted at the men’s college, of course it was. And it was sort of hushed under the carpet, you know, so long as the exam results keep coming through, everything will be alright. So it just sort of, you know, passed over. We, I had a group of about six of us who used to meet every night together usually in my partner’s room because she had the free gas fire … And, one of them was a bit more savvy than the rest of us. And I sort of confided in them that me and my friend were you know, were thinking about our futures as a relationship and one of them said ‘You’re the most obvious lesbian I’ve ever known!’ and I said ‘Obvious what?’ and she said ‘lesbian’—I had never heard the word I had no idea what she was talking about and I had to ask ‘well, what does that mean?’ and she said ‘well women who fall in love with women’ and at the age of 21 I had no idea, just no idea. But the fact that there was a word for it, meant there must be others, you know. So I sort of began at that stage to think, well it wasn’t actually necessary to change sex, there might be other people too.”

The reflections in the above transcript touch upon a range of core themes which historians of sexuality have drawn upon. In many ways, this demonstrates the value of micro(oral)histories. That while they may not be always representative of wider social currents, because they are by nature an individual’s own story, they often provide more nuanced picture of those wider social phenomena. Language is a core component of such social understandings and Cynthia’s description of discovering the word “lesbian” in this context provides one example of the ways that language shifts, transforms and alters across queer history, and how important affirmative terms and identities can be.^8^

The areas of sexuality and masculine dress for women has been an area of debate among queer historians. Doan (2006) argues that women dressing in a more masculine manner in the post war years was not at the time necessarily indicative of lesbian identity/behavior, such dress was viewed as a reaction to the war, not as a sign of sexual deviance. Doan (2006, 2013) argues that there was not a panic over the increase of “masculine” women *per se*; their appearance was more humorous and a novelty. In contrast, a number of lesbian historians have identified such “cross-dressing” in this period as distinctly queer. For example, working-class women “passing” as men in order to gain work; to marry their lovers and to gain other privileges given only to men (Duberman et al., 1989; Jennings, 2007a, 2007b; Newton, 1989). Charlotte Wolff, a queer Jewish doctor who later knew Cynthia personally, was arrested by the Gestapo precisely for “dressing like a man” during the Second World War which prompted her to flee Germany (Brennan & Hegarty, 2010; Hubbard, 2020). Such comparisons of dress style and sexuality are equally bounded by gendered ideas of clothing and conflations of sex and gender.

In the above reflections, Cynthia described how initially she felt she would have to change sex in order to eventually marry the woman she was in a relationship with. In response to such a revelation, she also sought the help of her family and their doctor. This provides one small example of not only the medicalization evident in queer history but also the entangled nature of LGBTQ histories (see Hubbard & Griffiths, 2019). The concepts of sex, gender and sexuality are not as distinct as they might appear and such understandings have dramatically changed across the 20th century. In many ways, Cynthia describes the responses to her coming out as supportive. This is somewhat in contrast to dominant narratives of pathologization and stigmatization of this time. Generally, queer women received less criminal, medical and psychological attention than gay men, whose sexual activities were criminalized and their sexualities directly pathologized (Dickinson, 2015). However, that is not to say that there was no medico-scientific gaze upon women (see Carr & Spandler, 2019).

Indeed, the romantic and/or sexual relationships between women were sometimes framed as other sorts of relationships. Cynthia here touched upon the “school crushes” and “romantic friendships.” Of course, there remain varied ideas about relationships and intimacy, and both “romantic friendships” (Donoghue, 1993; Faderman, 1981, 1991; Jennings, 2007b) and “school crushes” (Jennings, 2007a, 2007b; Vicinus, 1989), suggest that such relationships were not necessarily sexual. Yet, it’s worth highlighting how some women framed in this way have, at a later date, had further evidence revealed that shows unexpected sexual relationships—Anne Lister is perhaps the most prominent example in British lesbian history. By framing romantic and/or sexual relationships between women as friendship alone, queer women’s stories become obscured and hard to unearth. Such erasure is not only challenging from a historical perspective but as evidenced in the above, keenly felt by individuals like Cynthia. Later, it was the hidden nature of lesbian sexuality that moved her to do something to fight against the isolation of lesbian women after she had moved to London with her girlfriend from Cambridge in the early 1950s.

## 1960: The Minorities Research Group and Kenric

After University, in 1957, Cynthia moved to London where she has lived ever since and “never wanted to leave.” In London, she initially struggled following the breakdown of the relationship and soon realized how difficult it would be to meet other lesbians.

“I think the realisation that the relationship was ending and that I had no one else and no means of meeting anyone else. Whereas other people could go to, I dunno, discos or dances, or hobby groups or whatever and meet partners and say ‘would you like to come out for a coffee,’ you know, you can tell people you fancy them and you’d like to get to know them better and if you’re a homosexual, there’s no sort of group that you could go to … So you start off on a level playing field knowing that you’re all gay, though we didn’t use the word gay much in those days … I felt very strongly that there should be groups, some organisations but there wasn’t.”

Cynthia’s distress culminated in her seeking out professional psychological support. However, unlike many queer people of this era who experienced substantial violence in the face of coming out to those working in the psych disciplines, Cynthia was actually supported by those she had contact with. In fact, her psychiatrist put her in contact with other lesbian women and introduced her to the lesbian club The Gateways (see Gardiner, 2003; Jennings, 2006 for further details). Such accounts go further to illustrate the complex history of the psy disciplines with queer people (see Dickinson, 2015; Hegarty, 2017; Hubbard & Griffiths, 2019; Spandler & Carr, 2020).

“ … because of my emotional state, I’d been having psychological, psychiatric treatment of a sort and I’d had antidepressants I was put in touch with a psychiatrist, who lived in Chelsea, as privately, she didn’t charge. She was an eccentric woman who used to go off cycling around Marrakesh and places like that. And I don’t know why, she was … she wasn’t gay, but she was a bit eccentric.… And she was quite helpful and she said had I heard of The Gateways, and she’d heard about it and she knew where it was, and she arranges for me to meet one of her other clients who was a member to take me down there one night. So I met up with this completely strange woman, I was taken down to The Gateways and I came home and was sick [laughter]. Because it was, I was a frantic mess from it all. Yes, I mean the whole emotional thing and so I came home and vomited.”

**Katherine**: So all, the all the experiences you had with doctors and psychiatrists and things, were they always relatively neutral or ok about … ^9^

“Yes there was nothing I would say that was harmful. The family doctor did his best but couldn’t anything very positive. The psychiatrist I was put in touch with did her best, at least she got me a contact with someone who could take me to The Gateways club and say, ‘this is what’s available’ you know. But I suppose because I felt an outsider in a way that all these people down there dancing, they all knew each other and I was sitting in a corner [laughter] so go home and be sick was my reaction.”

**Katherine**: Did you keep going back?

“Yes I did go back. I sort of pulled myself together and thought if you want to do anything you’ve got to get there and get out and do things. Yeah.”

**Katherine**: So what was it like there?

“It was very noisy. I’d never been to clubs or discos at all. Dancing wasn’t really my thing … No, it wasn’t my sort of scene but at least you were able to sit in a corner and chat to people if you wanted so I met one or two quite interesting people. From different backgrounds. And some of them I got quite friendly with.” “ … there was definite butches and definite femmes. And I think Julie [Switzer]^10^ and I when we started, went down there together were unusual in that we might both be wearing collar and ties and sweaters and sort of both fairly butch. And that would be unusual. Because most couples would be a femme and a butch and to have two butches would seem a bit unusual so we were not following the normal pattern … I was aware that that was not the norm.”

Cynthia’s account of The Gateways and butch/femme dynamics is very similar to others who have accounted for lesbian culture in this postwar period (Gardiner, 2003; Hamer, 2013; Jennings, 2007a). Prior to her full integration within this scene, Cynthia made effort to find more contact with others like her. Cynthia was inspired to join the Homosexual Law Reform Society.

“So what I did then was, there was something called the Homosexual Law Reform Society, that was the only thing I knew about. So I contacted Anthony Grey who … the solicitor, who was the secretary of the association. And women could join, there was no reason why women couldn’t join. Though the law didn’t apply to them at all.”

Cynthia was keen to facilitate more social contacts via this society, however this was not possible immediately.

“Anthony was a very very cautious chap, being a lawyer himself, I suppose he was very aware of the law and the ramifications of arranging any kinds of meetings. So that their meetings were very strict and organised, in public halls, there was no sort socialising and I felt very strongly that people wanted to socialise … . I offered several, on a couple of occasions at least, to have coffee mornings in my own flat, you know on the premises so that people could just come to and have a chat and he wouldn’t even accept that. That that was potentially getting people together and I would be colluding- that I’d be an accessory for these men to have sex.”

Spring boarding from this initial contact, in an effort to create more social connections in 1963 she and four other women established the Minorities Research group: the first known official lesbian or “female homosexual” group in Britain.

“ … eventually he [Anthony Grey] did kind of accept that I was, partly determined to do something and he eventually put me in touch with Chad Varah who was the founder of the Samaritans because Chad Varah was particularly interested in the prosperity of lesbian groups because the law didn’t cover them … and through him met one or two lesbians that he knew and got together with them to finally start the Minorities Research Group. There was only five of us you know right at the start.”

Alongside Cynthia, the five founding members were Esme Langley, Diane Chapman, Julia Switzer (who was Cynthia’s partner at the time) and Paddi Dunkley:

“ … [Esme] had literary aspirations, I don’t think she was a particularly good writer herself but she would have liked to have been the editor of an international literary magazine aspiring really high. Her and her friend Diane Chapman,^11^ that was their motivation basically. She wanted to start a magazine. I wasn’t particularly interested in a magazine my interests were more in social contact.”

“Yes Julia Switzer she was my friend who I had met through Chad Varah, yes.”

“Yes Paddi Dunkley, she disappeared completely, we lost touch with her, I think before Kenric started even, she disappeared and I don’t know what happened to her, she just kind of vanished.”

Together in 1963 they met in Langley’s flat in London for the first meeting. Langley was the somewhat dominating force behind the group and their publication *Arena Three*, though her dominance soon caused issues within the group. Membership was quite open, though initially married women required their husband’s permission to join. The Minorities Research Group stressed: “all those prepared honestly to endorse these aims and objects were admitted to membership, whether their interest was directly personal or sociological, or from disinterested good will.” Their more open membership was directly related to their intention to “free female homosexuality from the prurience, sensationalism and vulgar voyeurism with which it is associated in some minds” (see Hubbard, 2020; Jennings, 2007a).

The Minorities Research Group outlined their objectives as follows:

1. To provide a center wherein homosexual women can meet others for discussion of their differing views, problems and interests. It is now becoming generally recognized that isolation is a potent factor in inducing neurosis.
2. To provide material for medico-social research workers and writers who wish to investigate the condition.
3. To seek ways of improving the public image of the Lesbian by familiarizing this fairly common condition, and of removing from it the aura of social stigma.
4. To publish and circulate monthly to members the magazine *Arena Three*, in which items of particular concern to homosexual women can be discussed, but which will also publish material of more general interest.
5. To arrange meetings, debates, lectures and conferences and to promote intelligent and properly informed press and radio comment in relation to this minority group.

(see Hubbard, 2020; Jennings, 2007a for full details and history of the Minorities Research Group). Cynthia remembers this early development of the group and highlights her particular emphasis earlier on about socializing being in contrast with the publication interests of Langley.

“It was a case of the five of us getting together and publishing [*Arena Three*] [laughter] … I didn’t have a lot to do with to be honest. That wasn’t my interest, I wanted to start a social group where people could meet. Esme wasn’t in favour of that, she didn’t particularly want people to meet she just wanted to produce a magazine, so basically it was a case of putting the magazine together, people subscribe the magazine so they sent money in to be subscribers, which meant they got sent the magazine by post. But there wasn’t much in the way of meetings for quite some time as far as I remember.”

The name selected for the group was also chosen because of its assumed respectability and neutrality (see also, Hubbard, 2020; Jennings, 2007a; Tooth Murphy, 2020a).^12^ Cynthia recalled:

“ … to be honest I don’t, when this came up with the title. Minorities was chosen as a word which sort of didn’t include the word gay or homosexual or anything like that. Research was to give it respectability. [laughter]. There wasn’t really any sort of real commitment to research on minorities or homosexuality. We just had to think of a title that was neutral, didn’t arouse suspicion, from people who wanted to be secretive.”

The Minorities Research group did not only supply monthly copies of *Arena Three*. Soon after getting established monthly pub meet ups began, just as Cynthia had hoped. Here, they held debates, discussions, and lectures, as well as providing a social environment for women often isolated and struggling with social stigmatization, though these were mainly based in London. Following the burst of membership and the constant stream of letters to headquarters, it quickly became apparent that additional resources were needed for many of the women seeking their help. In *Arena Three*, the committee members reported that they had been inundated with requests for information, help and advice from all sorts of people including lesbians or “women who fearfully suspect themselves of being lesbian.” Personal counseling services and postal correspondence to individuals became key work and this was apparent across a number of “homophile” groups at this time. The Minorities Research Group became more known, despite advertising issues, and soon became somewhere for health and social services to direct queer women to. The demand for the network and supportive friendships afforded by the Minorities Research Group demonstrated their argument that such an organization was essential in order to counter the isolation, fear and worry of many, especially remote, lesbian women.

However, soon after its establishment, in 1965, one of the Minorities Research Group geographical sub-groups- the Surrey and South West London group—became “disquieted with its running by Esme Langley” (Hamer, 2013; Hubbard, 2020; Jennings, 2007a, 2007b; Oram, 2007). In particular, there were concerns around the intertwined finances of the Minorities Research Group and Langley’s personal accounts. She had also registered *Arena Three* as a public limited company in her own name and the timing of this tension coincided with the breakdown of the relationship between Esme Langley and Diane Chapman. This new group decided on the name Kenric (joining Kensington and Richmond, the main locations within its geographic grouping). More importantly, however, Kenric wanted to be more democratic and provide greater social opportunities for lesbian women. Cynthia was a member of this splinter group, and is to this day is connected to Kenric. It is now the longest running lesbian organization in Britain. And both these groups are considered pivotal in the history of lesbian liberation in Britain (Hamer, 2013; Hubbard, 2020; Jennings, 2007a, 2007b; Oram, 2007).^13^

“ … there was one disastrous evening when my father died … there was a clash as far as I remember at The Gateways club with the bookings where the MRG meeting was at The Gateways at the same time as the potential Kenric wanted to meet there. And so the Kenric people [including Cynthia] agreed to just go and leave MRG to it. And that was the night my father was killed in a road accident and the phone call came through to The Gateways club because my mother knew that’s where I was [supposed to be]. … so I wasn’t able to get the message that my father was in hospital after a road crash. Whether that was the night that Kenric started or whether it was a separate night I’m a bit vague about that. Obviously I would have been very emotional at the time. I kind of held it against Esme for quite a long time that I didn’t get the message about my father’s accident but it wasn’t her fault. But anyway, whenever it was, whatever it was about that time, the people in Kenric who wanted to have a proper constituted organisation, preferably nation-wide but at least starting in the London area, those of us. Though I wasn’t there at the initial meeting when they decided to do it.”

“[Kenric] was very formalised, properly written constitution … So it was all set up with that kind of structure.” “I served as chairman certainly for one year, I don’t think I served in any other capacity. But yes I was always an active member. I never had aspirations to be at the, sort of forefront of it. I just wanted to know that the organisation was there … But you know we did things like organising a library, read a lot of books, that sort of things and they were kept at my house as we had central premises, Julie and I. And both our mothers helped to run the library.”

Cynthia’s mother actually had quite an affirmative role within the group:

“There was only a few people who were out to their families in those days, very few. My mother would spend hours at the, if we went to meetings, pub meetings or whatever, library meetings, general meetings, would spend hours being talked to by members who wanted to talk to a mother, you know because they wouldn’t talk to their own mothers.”

**Katherine:** So your mum would come along to things?

“Oh yes, yeah. They would pour their hearts out to mum who would you know listen to their heart breaking stories. It was quite upsetting for her I suppose in a way. To hear all their difficult times other people had with their families. So I was so so lucky in that sense that I didn’t have that problem.”

Hoping to help others who were not as lucky to have a supportive family, or such a strong sense of self, Cynthia stepped in to a supportive role which adopted more psychological aspects.

“I did what you might call counselling, a counselling service through Kenric to anybody who was having emotional problems like I had gone through. Hopefully to be able to help them and just have a chat really. I didn’t have any psychiatric qualification but I was perfectly prepared to have people come and talk to me, you know like they talked to my mum at Kenric meetings. Just somebody to talk to and give whatever counselling help I could.”

**Katherine:** Did many people take you up on that?

“Quite a few, yes, yes. And I hope I was helpful to some. There was some, funnily enough the one that I remember was one I was completely unable to give any help to at all.”

Across Cynthia’s own account of her life from the 1960s there are some aspects which correspond to previous historical literature, but also some aspects that challenge certain narratives. Her own story matches well with other accounts of the establishment of the Minorities Research Group, The Gateways and the work of the Homosexual Law Reform Society, which in many ways represent the British counterparts of the so-called “homophile” movement (see Hubbard, 2020; Hamer, 2013; Lewis, 2013; Jennings, 2007a; Oram, 2007). Her descriptions of The Gateways and butch/femme dynamics is also reflective of wider lesbian culture in this postwar period (Davis & Lapovsky Kennedy, 1986; Jennings, 2007a), as are her accounts of the isolation and community felt within these spaces are also reminiscent of those who were in similar circles (eg, Gardiner, 2003; *Inventing ourselves: Lesbian life stories*). Yet, there is also some affirming and positive stories that Cynthia accounts which, while not uncommon, are in contrast to the dominant narratives. These stories provide important nuance and complexity to such histories.

One key area where Cynthia remembers kindness and support is from both her own family and from the medical professionals she sought help from. Generally at this time, Psychology and medical professionals in Britain were more focused on the pathologization and “treatment” of LGBTQ people.^14^ Her experience therefore lends weight to arguments that highlight the complexity within the histories of Psychology and LGBTQ people. That is not, of course to diminish the many instances of systematic violence enacted upon queer people in the name of science and the psy-disciplines specifically. For example, the introduction of “homosexuality” as a mental disorder; the use of inversion therapy/conversion therapy; the (lack of) response to the HIV/AIDS crisis (see Davison, 2021; Dickinson, 2015; Hegarty, 2017; Hubbard & Griffiths, 2019). Instead, Cynthia’s experience shows the value of individual stories in illustrating a multifaceted period of queer history, which echoes others who had similar varied experiences. Cynthia also described how Psychology (in the form of counseling) was also used by community groups such as Kenric and the Albany Trust,^15^ the charitable component of the Homosexual Law Reform Society, and so was not rejected wholesale by queer organizations (see Hegarty, 2017; Hubbard, 2020). The support that Cynthia describes herself and her mother providing demonstrates the value of community for marginalized groups and I argue, how pivotal Cynthia was in creating this for many queer women in Britain.

## 1970s onward: Quieter activism

During this period of forming Kenric, and alongside her full-time IT work, Cynthia also completed a degree in Psychology at Birkbeck from 1965 to 1969. Following a hiatus of 25 years, she also reestablished her passion for flying in the 1970s. Throughout her time with the Minorities Research group and establishing Kenric, she had engaged with public events which were arguably instrumental in lesbian liberation action. She explained that in the 1970s, once the Gay Liberation Front had become active, she felt there had been a substantial shift is visibility and people’s ability to come out.

“I talked to Universities and television interviews and radio interviews and that sort of thing so I felt I had done what I could. I’m not a very physical sort of person, going on marches isn’t really my thing. I was worried about where the nearest toilets were [laughter]”

**Katherine:** What interviews or radio shows or television talks did you do? What kind of things did you do?

“I did one or two University [talks]. To student unions that sort of thing. I think there was a couple in Birmingham, one in Coventry, the midlands mainly. There might have been one or two in London and then I’d had television interviews, there was one with Brian Magee^16^ and I think there was a radio interview as well. Things like that. I was quite happy to do that, that didn’t involve much effort, physical effort. I’ve always been a lazy person. Mental effort, I guess more. That’s more my thing.”

“I’ve never marched; I’ve never been into that sort of thing. It came later, it sort of followed on. And by [that] stage I was satisfied that people could be as open as they wanted or didn’t want and that you know I achieved that, that was my goal.” “I think Gay Lib would have happened even without Kenric, I don’t think Kenric started Gay Lib. Gay Lib [was] going to come along anyway so I can’t claim that, you know we started something [that] wasn’t going to happen anyway. I think the general society was changing and that would have happened.”

The Gay Liberation Front, despite only lasting two years, remains a highly significant point in queer British history for its public demonstrations and activism (Weeks, 2016). Nonetheless, other forms of activism remain important and some of the work that Cynthia contributed to, and the individual stories of support and kindness, of connectivity and community, are often lost in louder histories of activism. It is true, that Cynthia does not consider herself an activist. Her work here was of a quieter nature; she did radio shows, talks at Universities and a TV interview. This “mental effort” as she calls it might not be quite as visual as marching in the streets but it nonetheless has had a profound effect on lesbian history in Britain (see also Oram, 2007).^17^ I therefore argue that this *is* activism—she helped build a community. While the Minorites Research Group had varied approaches at the outset, it is likely that both the social aspects and the publication of *Arena Three* were important activist actions to alleviate the difficulties of queer women at this time. Cynthia argues that gay liberation was on its way regardless, but it is clear that the work she put in especially in the 1960s made those efforts from the 1970s at the very least, more possible.

Following this work, Cynthia then became Assistant Director in Social Services and helped set up the service Dial-a-Ride in the 1980s.^18^ After semi-retirement, she worked as a driver for them (“the first job I really loved doing”) for another 15 years. In drawing more toward the present day, Cynthia reflected that her work in setting up Kenric perhaps had the most impact, though she argues that its purpose and necessity today is very different to that of the late 1960s. However, the importance of an active and rich social life remains just as vital to Cynthia now-a-days as it did when she first aimed to set up a lesbian organization in the 1960s. She continues to value her ongoing independence and friendships. It is clear from Cynthia’s account of her life that infectious confident happiness and extroverted love of socializing have endured throughout the decades. She described herself as “contented” and just enjoying her life. This much is clear from meeting her, as she kindly shared her life, her home and her Pepsi max with me for several hours over the interview. Yet, her description of her life as “fairly straight forward” is one area where we might disagree. As this micro(oral)history shows, Cynthia has led a fascinating life so far and her individual story can contribute to wider narratives of British lesbian history.

## Conclusion

At the time I met Cynthia in the beginning of 2019, she was 84 and vivacious. It is only recently that Cynthia made the decision to stop flying altogether after being informed the air pressure could exacerbate an existing eye problem. She has a thriving social life, including still going to the pilot’s club house. She laughs it off when I highlight how she has paved the way for many women, myself included, to live in the ways that they do. I experienced her as distinctly humble, fun, independent, and honest.

In many ways, Cynthia’s account of her life here reflects much of what lesbian historians have demonstrated through various works but from a brand new perspective. Her account therefore reinforces such analysis (see Gardiner, 2003; Hamer, 2013; Hubbard, 2020; Jennings, 2007a, 2007b; Oram, 2007), but also provides a nuanced, individual narrative and her own personal story. Such personal stories demonstrate the idiosyncrasy of experience and contribute to the preservation of queer history, which is why oral histories such as this, as well as those in the Hall Carpenter archive, are so vital.^19^ Arguably, Cynthia’s own account here shows the distinct nature of various forms of British activism. Certainly, the actions of the Gay Liberation Front are of vital importance for this history, but the quieter activism in the decade previous still made substantial headway (Oram, 2007).

This micro(oral)history provides a history spanning decades in Cynthia’s own words and such information can be used to (re)consider and reflect upon the wider cultural historical context. I argue that the Cynthia’s oral history provides a fruitful addition to understanding broader mid-century queer British lesbian history. This article therefore presents not only aspects of my analytical thinking of her oral history but provides future queer researchers with a resource from which they too can draw upon. It also challenges some dominant narratives about the psy-disciplines within queer history by showing the multiple and varied ways in which Cynthia engaged with professionals and utilized counseling herself. Finally, I argue that as a single oral(micro)history this article shows the power of community; and provides an in depth personal and unique perspective on such community at a vital point of lesbian history in Britain.

Despite meeting Cynthia in 2019, decades after her key involvement with the Gateways, the Minorities Research Group and the setting up of Kenric, I was a little surprised to find just how easy it was to imagine Cynthia doing exactly the things she described. She learned to fly at aged 18, went to Cambridge as one of the first and only women in engineering, and was pivotal in setting up the very first known official lesbian organization in Britain. Yet, she remains so close to the woman she described from the past. She still has a prevailing masculinity, a sense of energy and confidence. Upon meeting her it is easy to see how she, someone so vivacious and quick to laugh made so many people less lonely, less isolated and more confident in themselves. I left her home with a smile on my face and a biscuit in my hand.

## Notes

1. This interview was made possible by Kenric, who kindly forwarded Cynthia’s contact details to me with her permission. Initially, I contacted Cynthia as the copyright holder to ask her to grant me permission to use an *Arena Three* image in Hubbard (2020). She kindly allowed me to use the image and later agreed to be interviewed. The interview followed a largely unstructured format and was conducted at Cynthia’s home in March 2019. The interview lasted 1.5 hours and was conducted in a single sitting. All unattributed quotes in this article are from this interview. Full ethical approval was received from the University of Surrey.

2. See Boyce, Engebretsen, Gonzalez-Polledo, and Posocco (2017), Boyd & Ramirez (2012), Clarke and Hopkins (2002), Davis and Lapovsky Kennedy (1986), Dickinson (2015), Hegarty and Chase (2000), Summerskill (2013), Traies (2018), see Boyd (2008) for discussion of oral history and queer theory in US- focused histories.

3. In line with such feminist principles, Cynthia was invited to be a coauthor on this article but declined the offer. She was supplied the original draft of the article to ensure she was happy with it and was able to request any changes she wished. She has also been provided with this final version to approve prior to publication.

4. Now Leeds and Bradford Airport.

5. This actually occurred from 1948.

6. Terminology and understanding of the word “crush” has changed somewhat and these sorts of relationships at school have been undergone particular analysis by queer historians, for example, see Vicinus (1989, see also discussion by Banner 2003; Faderman, 1981).

7. It is worth noting that while this was the case for the vast majority of family doctors in England at the time, it is not necessarily reflective of medical science as a whole which has with specialisms grounded in sexology from the 19th century (see Gill-Peterson, 2018; Snorton, 2017; Styker, 2017 for detailed trans histories across this time period).

8. In recognition of the various ways language changes, it is worth highlighting my own use of “queer”, in itself another example of a dramatic shift in usage and meaning. Queer is utilized at points throughout the paper precisely because of its reclaimed status and its inclusivity (I have explored the use of “queer” in historical writing elsewhere: Hubbard, 2017, 2020).

9. Cynthia had numerous interactions with medical professionals, all of whom were relatively affirming, or at the very least did not treat her as if she was pathologically unwell solely because of her sexuality. She also was aware of medical interventions around homosexuality at the time: “I was aware of things like aversion therapy, you know which I thought was totally wrong. But, but, I never knew anyone who had undergone it… didn’t really impinge upon me very much.” However, she described more positive experiences with medical professionals. One such professional she knew was Charlotte Wolff, a German born Jewish psychotherapist who, as a queer woman herself worked alongside and with activist groups at this time including the Minorities Research Group, Kenric, and the Albany Trust (for more information on Wolff, see Brennan & Hegarty, 2010, 2012; Hubbard, 2020). What was most prominent in Cynthia’s memory about Wolff was her pet snake, a fascinating fact which is absent from other records of Wolff:“Oh yes Charlotte. She had a python. It made me nervous yes. It used to lie on the couch behind us. [laugher] I was never very happy about that. It was quite a big python.”“Mainly I was concerned about the python to be honest. [laughter] That great thing. She assured me it was well fed. [laughter].”“It was called Pythagoras by the way.”

10. Julie Switzer was one of the other founding members of the Minorities Research Group and Cynthia’s partner at the time.

11. Esme Langley and Diana Chapman were in a relationship the beginning of the Minorities Research Group (see Jennings, 2007a and *Inventing Ourselves Lesbian Life Stories*; 1989).

12. Relatedly, though the Minorities Research Group included both working and middle-class women, the majority of members were middle-class and this was particularly distinct in the ethos of *Arena Three* (see Hamer, 2013; Hubbard, 2020; Tooth Tooth Murphy, 2020a).

13. The Minorities Research Group continued until 1971 when Esme Langley emigrated. Other members of the continued its tradition and set up Sappho which continued until 1981. See Oram (2007) for details about the later years of the Minorities Research Group and Tooth Murphy (2020a) for a recent analysis of *Arena Three*.

14. See Davison (2021); Dickinson (2015), Hubbard (2020), Hubbard and Griffiths (2019), Jennings (2008) and Spandler and Carr (2020), for further exploration about the historical relationships between Psychology/Psychiatry and queer communities at this time.

15. With whom Charlotte Wolff also worked.

16. Bryan Magee had worked with the MRG to produce a television programme as a part of the *This Week* current affairs programme in 1964, which included him being allowed to attend a MRG meeting. He later, however, made assertations in his journalistic writings that angered the Minorities Research Group (see Jennings, 2007a).

17. The Minorities Research Group had some interaction with the Gay Liberation Front and the women’s liberation movement more broadly at this time (Oram, 2007).

18. Dial-a-Ride is a London based door-to-door transport service for members with long-term disabilities.

19. A selection of the Oral Histories available in the Hall Carpenter collection are also available as a book *Inventing ourselves: Lesbian life stories*. Likewise, documentary materials relating to this history such as those from: the Gay Liberation Front, the Albany Trust, the Minorities Research Group, Kenric, the Homosexual Law Reform Society and others, are available at the Welcome Library, the British Library and the London School of Economics Library. Relevant collections can also be found at the Bishopsgate Institute (all London).
